# Changes in sexual-function scores after washed microbiota transplantation in men with sexual dysfunction: an exploratory single-arm pre–post observational study

**DOI:** 10.3389/fmicb.2026.1906680

**Published:** 2026-08-05

**Authors:** Junyi Chen, Jiansen Yuan, Dajiang Li, Caimei Lu, Lingui Huang, Ali Chen, Chuyan Zhong, Dehao Chen, Zhichu Qin, Xingxiang He, Lei Wu

**Affiliations:** 1 Department of Gastroenterology/Microbiota Medicine, Research Center for Engineering Techniques of Microbiota-Targeted Therapies of Guangdong Province, The First Affiliated Hospital of Guangdong Pharmaceutical University, Guangzhou, China; 2 Guangdong Provincial Key Laboratory for Research and Evaluation of Pharmaceutical Preparations, Guangzhou, China; 3 Center for Drug Research and Development, Guangdong Provincial Key Laboratory of Advanced Drug Delivery System, Guangdong Pharmaceutical University, Guangzhou, China; 4 Foshan Shunde Integrated TCM & Western Medicine Hospital (Foshan Shunde Junan Hospital), Foshan, China; 5 Dongguan Humen Traditional Chinese Medicine Hospital, Dongguan, China

**Keywords:** fecal microbiota transplantation, gut microbiota, male sexual dysfunction, sexual function, washed microbiota transplantation

## Abstract

**Background and aims:**

Current therapies for male sexual dysfunction (MSD) exhibit variable efficacy and potential adverse effects. Washed microbiota transplantation (WMT) is an emerging microbiota-based intervention, but its clinical relevance to MSD remains unclear. This exploratory single-arm pre–post observational study aimed to describe within-subject changes in sexual function, sexual quality of life, sex hormones, and gut microbiota profiles before and after WMT in men receiving WMT for established clinical indications.

**Methods:**

A total of 37 male patients receiving WMT were enrolled, including 16 patients with baseline MSD and 21 patients with normal baseline sexual function. All participants received WMT via the lower gastrointestinal tract, and no placebo, sham, untreated, or standard-care control group was included. Sexual function and quality of life were evaluated using the Sexual Life Quality Questionnaire (SLQQ) and the Arizona Male Sexual Experience Scale (ASEX), which were designated as the primary exploratory endpoint. Serum sex hormone levels and gut microbiota profiles characterized by 16S rRNA gene sequencing were defined as secondary exploratory outcomes. The primary sexual-function analyses were based on within-subject pre–post comparisons, and no formal power calculation was performed for the secondary hormone or microbiome outcomes.

**Results:**

In within-subject analyses, favorable changes in sexual-function scores were observed at one-month follow-up in the baseline MSD group: 10 of 16 patients no longer met the ASEX-defined MSD threshold, ASEX scores decreased, and SLQQ scores increased. However, because this was an uncontrolled single-arm pre–post analysis, these changes cannot be attributed causally to WMT and may partly reflect placebo or expectancy effects, regression to the mean, natural symptom fluctuation, concurrent care, or improvement in the underlying chronic conditions for which WMT was administered. The hormone and microbiota analyses were limited by the small number of patients with baseline MSD. Therefore, the non-significant hormone findings should be interpreted as inconclusive rather than as evidence of no endocrine involvement, and the microbiota findings should be regarded as exploratory and susceptible to both type I and type II errors.

**Conclusion:**

In this exploratory single-arm pre–post observational cohort, favorable within-subject changes in sexual-function and sexual quality-of-life scores were observed at one-month follow-up after WMT. However, because the study lacked a placebo, sham-procedure, untreated, or standard-of-care control arm, these changes should not be interpreted as evidence of therapeutic efficacy. The findings are hypothesis-generating and require validation in adequately powered randomized placebo- or sham-controlled trials.

## 1 Introduction

Normal sexual function constitutes an essential component of human health and reproduction. As a multifaceted phenomenon, sexual function is modulated by sex hormone levels, psychological factors, neurological regulation, and hemodynamic mechanisms (Forouzannia et al., 2012; De Berardis et al., 2007; Zesiewicz et al., 2003; Kendirci et al., 2007). Male sexual dysfunction (MSD) is common in the general male population, although prevalence estimates vary according to age, population, diagnostic criteria, and assessment tools. The National Health and Social Life Survey reported sexual dysfunction in 31% of men aged 18–59 years, while a population-based study among Chinese married men aged 30–60 years reported that approximately 15% had at least one form of MSD (Laumann et al., 1999; Zhang et al., 2013). MSD encompasses a spectrum of clinical manifestations, primarily including erectile dysfunction (ED), premature ejaculation (PE), hypoactive sexual desire disorder, retrograde ejaculation, and dyspareunia (Salonia et al., 2025).

Emerging evidence suggests a possible association between gut microbiota and MSD, although the direction and causality of this relationship remain uncertain. The gut microbiota—a complex ecosystem comprising bacteria, archaea, fungi, and viruses colonizing the intestinal tract—plays pivotal roles in maintaining human health through metabolic regulation, immune modulation, and neurobehavioral interactions (Chen et al., 2021; Hoyles et al., 2018). Comparative analyses reveal marked dysbiosis in the gut microbiome of ED patients versus healthy controls (Zhu et al., 2024). Such microbial imbalance, termed dysbiosis, correlates with chronic conditions including metabolic syndrome, diabetes, inflammatory bowel disease (IBD), neurodegenerative disorders, and male infertility (Kaltsas et al., 2023; ACEVEDO-ROMáN et al., 2024; Askarova et al., 2020). In ED pathophysiology, dysbiosis may exacerbate systemic inflammation, endothelial dysfunction, and hormonal dysregulation—key contributors to erectile impairment (Kaltsas et al., 2025).

Current management of MSD includes oral pharmacotherapy, hormonal treatment when indicated, lifestyle modification, and psychological counseling. For ED, phosphodiesterase type 5 inhibitors are recommended first-line treatments with established efficacy and generally favorable tolerability, although mild dose-related adverse effects and contraindications should be considered (Shin et al., 2023). In psychogenic or mixed ED, PDE5 inhibitors may still be effective, particularly when combined with psychological or psychosexual interventions (Atallah et al., 2021). However, current treatments remain largely symptom-directed, supporting further exploratory research into potential microbiota-related mechanisms.

Washed Microbiota Transplantation (WMT), an emerging therapeutic modality with demonstrated efficacy in select enteric disorders, operates by reconstituting intestinal ecological homeostasis through infusion of rigorously screened functional microbiota from healthy donors (Shi, 2020). For instance, a study using high-fat-diet-induced erectile dysfunction rat models observed significant erectile deterioration in HFD-fed rats; however, WMT from normal-diet donors failed to restore erectile function (Xu et al., 2025). Therefore, this study should be interpreted as supporting a possible association between gut microbiota composition and erectile-function-related phenotypes, rather than as preclinical evidence for a restorative therapeutic effect of WMT on erectile function. Furthermore, gut microbiota influences male reproductive health via sex hormone regulation and behavioral modulation. Experimental data indicate that specific Lactobacillus strains enhance murine testicular size, testosterone levels, and sperm counts—parameters intimately linked to sexual function. Notably, microbiota transplantation from these treated mice was reported to improve reproductive metrics in recipients, reinforcing gut-microbiota–genital axis interactions (JUHáSZ et al., 2024).

This exploratory study aimed to describe within-subject changes in male sexual-function scores, sexual quality of life, sex hormones, and gut microbiota profiles before and after WMT. Because all participants received WMT for established underlying chronic conditions and no placebo, sham-procedure, untreated, or standard-of-care control group was included, the study was not designed to evaluate the therapeutic efficacy of WMT for MSD. The findings should therefore be interpreted as hypothesis-generating observations regarding the gut microbiota–sexual health relationship.

## 2 Materials and methods

### 2.1 Ethical approval

This study was approved by the Institutional Review Board of the First Affiliated Hospital of Guangdong Pharmaceutical University, Guangzhou, China (Approval No. 2024-43). This study was designed as a real-world, exploratory single-arm observational pre–post cohort study, in which all participants received WMT for established clinical indications rather than specifically as an experimental treatment for MSD. No randomization, placebo intervention, sham procedure, untreated comparison group, or standard-of-care control arm was applied. Therefore, the study was designed to describe within-subject changes over time and was not intended to estimate the WMT-specific therapeutic effect on MSD. Written informed consent was obtained from all participants prior to enrollment.

### 2.2 Study population

*Inclusion criteria*: (1) Male patients aged 18–70 years (inclusive), capable of comprehending and signing written informed consent voluntarily; (2) Regular sexual activity (defined as ≥1 instance per month for ≥3 consecutive months) within the preceding 3 months; (3) No use of the following medications within 4 weeks prior to the study: phosphodiesterase type 5 inhibitors, antidepressants, androgen replacement therapy, or sex hormone-modulating agents; (4) Patients undergoing WMT at our institution for established clinical indications, including but not limited to functional gastrointestinal, inflammatory or immune-mediated, metabolic, neuropsychiatric, oncology-related conditions, or gout. (5) Comorbidities under stable control with no adjustments to therapeutic regimens within the preceding month.

*Exclusion criteria*: (1) Congenital or acquired genital anatomical abnormalities; (2) Severe organic diseases, such as uncontrolled cardiovascular diseases, severe hepatic or renal dysfunction, or neurological disorders (e.g., spinal cord injury, multiple sclerosis) resulting in sexual dysfunction; (3) Requiring chronic use of medications that may affect sexual function during the study period (e.g., beta-blockers, antipsychotics); (4) Inability to complete follow-up due to personal reasons.

To minimize confounding in this observational pre–post study, we restricted enrollment to patients with stable comorbidities and no recent treatment modifications, and excluded participants with recent exposure to medications known to affect sexual function. Nevertheless, because the study lacked a concurrent control arm, these restrictions could not eliminate placebo or expectancy effects, regression to the mean, natural symptom fluctuation, or the potential influence of improvement in the underlying diseases for which WMT was administered.

### 2.3 Donor selection

Candidate donors were preferentially recruited from long-term local residents of Guangzhou and currently enrolled university students aged 18–25 years. A standardized questionnaire was used to screen for a history of psychiatric disorders, infectious diseases, and major systemic diseases. Subsequently, all candidates underwent a comprehensive physical examination and laboratory testing, including complete blood count, screening for infectious disease markers, and stool pathogen testing, to exclude potential infectious risks such as HIV, hepatitis viruses, and enteric pathogens. Each stool donation was required to provide at least 50 g of fecal material. Stool morphology was assessed according to the Bristol Stool Form Scale, and only samples corresponding to types 3–4, namely formed soft stools or smooth, soft stools, were considered acceptable.

### 2.4 WMT preparation and administration protocol

The WMT protocol strictly adhered to the standardized methodologies outlined in the Nanjing Consensus (Shi, 2020). For microbiota preparation, fecal samples were homogenized at a 1:5 ratio (100 g feces mixed with 500 mL of 0.9% saline) to generate uniform suspensions. The mixture was processed using the GenFMTer automated microbiota isolation system, which operates under controlled hypoxic conditions (O₂ < 0.1%) to preserve anaerobic bacteria during the one-hour protocol. The final microbiota suspension was administered via the lower gastrointestinal tract (endoscopy-guided delivery). This study descriptively assessed within-subject changes in male sexual-function-related outcomes before and approximately one month after a single WMT course, which comprised daily transplantation of 120 mL washed bacterial suspension over three consecutive days. Baseline values were established using blood tests and clinical assessments conducted prior to the initial WMT course. Follow-up clinical and laboratory outcomes were assessed approximately 1 month after WMT.

### 2.5 Data collection

Patient data were extracted from electronic medical records, including demographic information (age) and comorbidities (hypertension, diabetes, smoking, and alcohol use), as well as laboratory test results upon admission: follicle-stimulating hormone (FSH), luteinizing hormone (LH), estradiol (E2), progesterone (P), testosterone (T), prolactin (PRL).

### 2.6 Definitions

The Arizona Male Sexual Experience Scale (ASEX) is a psychometric tool specifically designed to assess MSD. This scale comprises five items with total scores ranging from 5 to 30. A total score of ≥19, any single item score of ≥5, or scores of ≥4 on any three items may assist clinicians in diagnosing MSD (Mcgahuey et al., 2000).

The Sexual Life Quality Questionnaire (SLQQ) is a standardized assessment tool that quantifies sexual health-related outcomes across two dimensions: (1) Quality of Life (QOL) and (2) Treatment Satisfaction for Erectile Dysfunction (THX). This 16-item self-assessment systematically evaluates the impact of sexual function improvement on QOL and treatment adherence by integrating subjective feedback from both patients and their partners (Woodward et al., 2002).

In this study, MSD was operationally defined according to ASEX criteria, namely a total ASEX score of ≥19, any single item score of ≥5, or scores of ≥4 on any three items. Because ASEX assesses multiple domains of male sexual experience, including sexual drive, arousal, penile erection, orgasm, and satisfaction, this definition reflects global male sexual-function impairment rather than a subtype-specific diagnosis of ED, PE, or hypoactive sexual desire disorder. Therefore, we use MSD to describe the study population and outcomes throughout the manuscript.

### 2.7 Sample collection

Fecal and blood samples were collected from patients 2 days before each WMT session (at baseline and approximately one month after the first WMT treatment). Fecal specimens were stored in collection tubes containing DNA stabilization solution (Invitek, Germany). All samples were maintained at −80 °C until sequencing.

### 2.8 Microbiome analysis

Genomic DNA extraction from fecal samples was performed using the E. Z. N. A.® Soil DNA Isolation Kit (Omega Bio-tek, USA) in strict accordance with the manufacturer’s protocol. DNA purity and concentration were quantified using a NanoDrop 2000 spectrophotometer (Thermo Fisher Scientific, Wilmington, DE, USA) to establish quality control standards for subsequent analyses. The V3-V4 hypervariable regions of bacterial 16S ribosomal RNA (rRNA) genes were amplified with primers 338F (5′-ACTCCTACGGGAGGCAGCAG-3′) and 806R (5′-GGACTACHVGGGTWTCTAAT-3′), followed by verification of amplicon integrity via 1.5% agarose gel electrophoresis. Purified amplification products were subjected to paired-end sequencing (2 × 300 bp) on the Illumina MiSeq platform by Novogene Bioinformatics Technology Co., Ltd. (Beijing, China).

Raw paired-end sequencing reads were merged using FLASH (v1.2.11) and quality-filtered with FASTP (v0.19.6). Sequences were denoised and chimeras removed via the DADA2 algorithm within the QIIME2 (version 2020.2) pipeline to generate high-resolution amplicon sequence variants (ASVs). After quality control and chimera removal, per-sample effective reads (Nochime) ranged from 65,416 to 105,490 reads (median 80,692, IQR 74,322–87,665; mean ± SD 81,486 ± 9,043) across 62 samples. To ensure cross-sample comparability, the ASV table was rarefied to 4,000 reads per sample for downstream diversity analyses. Rarefaction curves indicated that Shannon diversity stabilizes rapidly at low sequencing depths, and the observed features curves show a steep rise followed by a marked deceleration with increasing depth, consistent with diminishing returns beyond early depths. We acknowledge that rarefaction at 4,000 reads may reduce sensitivity for detecting low-abundance taxa and therefore interpret low-abundance genera cautiously.

Downstream microbial community analyses were conducted using the Novomagic cloud platform developed by Novogene Bioinformatics Technology Co., Ltd. According to the platform description, Novomagic is a cloud-based microbiome analysis platform that supports 16S amplicon data analysis, including alpha-diversity analysis, beta-diversity analysis, PCoA, PCA, and NMDS ordination, group-level community structure comparison, taxon-level comparison, correlation analysis, and functional prediction. In the present study, the following modules were used: alpha-diversity analysis using Chao1, Shannon, and Simpson indices; Bray–Curtis distance-based PCoA for beta-diversity visualization; group-level community structure comparison; phylum-, family-, and genus-level taxonomic composition analysis; exploratory taxon-level comparison; Spearman correlation analysis between microbial taxa and clinical variables; and Tax4Fun-based functional prediction.

Tax4Fun-based functional prediction was used only as an exploratory inference from 16S rRNA gene sequencing data. No shotgun metagenomic sequencing, metatranscriptomic profiling, metabolomic analysis, microbial metabolite quantification, or host functional assay was performed. Therefore, predicted pathway-level findings were interpreted as hypothesis-generating signals rather than direct evidence of microbial functional activity or microbiota-mediated mechanisms.

### 2.9 Statistical analysis

Data processing was performed using IBM SPSS Statistics (v26.0) and GraphPad Prism (version 10.1.2). Normality assessment was conducted based on sample size: the Shapiro–Wilk test was applied to evaluate data distribution for datasets with *n* ≤ 50. Categorical variables were statistically described using frequency counts with percentage composition. Continuous variables are reported as mean ± standard deviation (SD) for approximately normally distributed data and as median (interquartile range, IQR) for non-normally distributed data. Categorical variables are presented as *n* (%).

For within-subject paired analyses, paired effect estimates were reported as mean paired changes with corresponding 95% confidence intervals, where applicable. Mean paired change was calculated as the follow-up value minus the baseline value. Therefore, negative changes in ASEX scores and positive changes in SLQQ scores indicated favorable changes. For sex hormone analyses, paired effect estimates were calculated among participants with complete paired measurements, and no imputation was performed for missing follow-up data.

ASEX and SLQQ score changes were defined as the primary exploratory endpoint. The Holm method was applied to the four within-subject ASEX and SLQQ comparisons. For exploratory subgroup analyses of SLQQ according to depressive and anxiety states, the four paired comparisons were also adjusted using the Holm method. Serum sex hormone analyses were treated as secondary exploratory outcomes, and *p* values were adjusted using the Benjamini–Hochberg false-discovery-rate procedure within each hormone-analysis family. Microbiome taxon-level comparisons, predicted functional pathways, and microbiome–clinical correlations were exploratory.

Given the clinical heterogeneity of the WMT indications, exploratory multivariable sensitivity analyses were performed for changes in ASEX and SLQQ scores. Change scores were calculated as follow-up minus baseline values. Separate ordinary least-squares regression models were fitted for changes in ASEX and SLQQ scores using heteroscedasticity-consistent HC3 standard errors and t-based inference. Each model adjusted for the corresponding baseline score, age, baseline depression and anxiety scores, comorbidity burden, and indication category. In these models, adjusted mean changes and *β* estimates were reported with 95% confidence intervals. The β coefficient represented the adjusted difference in change between patients with functional gastrointestinal disorders and those with other WMT indications. All multivariable analyses were exploratory and were not intended to eliminate unmeasured confounding or establish causality.

## 3 Results

### 3.1 Patient characteristics

This study enrolled a total of 37 male patients aged 18–70 years. Among the participants, 21 individuals were classified as normal sexual function group, while 16 patients were classified into the MSD group (Table 1).

**Table 1:** Baseline characteristics of patients.

Baseline characteristics	Normal sexual function group (*n* = 21)	MSD group (*n* = 16)	*p*
Age (years)	49.86 ± 12.76	49.06 ± 13.57	0.86
BMI	23.20 ± 2.44	22.05 ± 4.15	0.34
Sexual frequency (times/month)	4 (1–8)	1 (0–2)	<0.01
Sexual duration (minutes)	7.5 (7.5–12.5)	2.5 (2.5–7.5)	<0.01
Comorbidities			
Type 2 diabetes (%)	3 (14.29%)	4 (25.00%)	0.44
Hypertension (%)	4 (19.05%)	2 (12.50%)	0.68
Alcohol abuse (%)	0 (0%)	1 (6.25%)	0.43
Smoking history (%)	3 (14.29%)	3 (18.75%)	1.00
Sex hormones			
Estradiol (pg/mL)	109.96 ± 47.36	80.02 ± 41.11	0.13
Prolactin (mIU/L)	235.52 (203.42–468.01)	309.3 (283.55–358.06)	0.65
Luteinizing hormone (IU/L)	4.23 (2.93–4.96)	4.13 (2.13–6.51)	0.80
Follicle-stimulating hormone (IU/L)	4.76 (3.32–7.32)	4.83 (2.78–10.73)	0.927
Testosterone (nmol/L)	22.28 ± 5.82	19.95 ± 10.82	0.651
Progesterone (ng/mL)	0.32 (0.32–0.52)	0.34 (0.32–0.53)	0.927

Categorical variables are presented as *n* (%), with percentages calculated using the number of participants within each group as the denominator. Continuous variables were compared using independent-samples *t* tests or Mann–Whitney U tests, as appropriate. Categorical variables were compared using Fisher’s exact test.

### 3.2 Patient enrollment characteristics

Among the 37 patients receiving WMT in this study, functional bowel disorders constituted the primary treatment indication (19 cases, 51.35%), including irritable bowel syndrome (11 cases, 29.73%), functional diarrhea (3 cases, 8.11%), functional dyspepsia (3 cases, 8.11%), and functional constipation (2 cases, 5.40%). Other treatment indications were distributed as follows: oncology-related conditions (4 cases, 10.80%), comprising patients with gastrointestinal symptoms and/or malignancies (2 cases, 5.40%) and chemotherapy-associated diarrhea (1 case, 2.70%); Crohn’s disease (2 cases, 5.40%); atopic dermatitis (2 cases, 5.40%); type II diabetes mellitus (2 cases, 5.40%); urticaria (1 case, 2.70%); rectal ulcer (1 case, 2.70%); and other conditions (6 cases, 16.22%), including amyotrophic lateral sclerosis (4 cases), gout (1 case), and depressive state (1 case) (Table 2).

**Table 2:** Primary diagnoses of patients undergoing WMT.

Primary diagnosis	Number of cases	Percentage (%)
Irritable bowel syndrome	11	29.73%
Functional diarrhea	3	8.11%
Functional dyspepsia	3	8.11%
Functional constipation	2	5.40%
Oncology-related conditions	4	10.80%
Crohn’s disease	2	5.40%
Atopic dermatitis	2	5.40%
Type II diabetes mellitus	2	5.40%
Urticaria	1	2.70%
Rectal ulcer	1	2.70%
Other	6	16.22%

The cohort was clinically heterogeneous, and WMT was administered for underlying chronic conditions rather than specifically for MSD. These primary indications may independently influence both sexual function and gut microbiota composition through gastrointestinal symptom burden, systemic inflammation, metabolic or endocrine abnormalities, psychological distress, medication exposure, and overall health-related quality of life. Therefore, microbiome differences observed in this cohort may partly reflect indication-related or disease-specific dysbiosis rather than MSD-specific microbial features.

### 3.3 Within-subject changes in sexual-function scores from baseline to one-month follow-up

None of the participants with non-MSD status at baseline met the ASEX-defined MSD threshold at one-month follow-up. Among the 16 patients with baseline ASEX-defined MSD, 10 patients (62.50%) no longer met the ASEX-defined MSD threshold at follow-up, whereas 6 patients remained classified as MSD. These paired status transitions are summarized in Table 3.

**Table 3:** Paired transitions in ASEX-defined male sexual dysfunction status from baseline to one-month follow-up after WMT.

Baseline ASEX-defined status	MSD at follow-up, *n*	Normal sexual function at follow-up, *n*	Total, *n*	Transition proportion	95% CI	Exact McNemar *p*
MSD at baseline	6	10	16	10/16 (62.5%) no longer met MSD threshold	35.4–84.8%	0.00195
Normal sexual function	0	21	21	0/21 (0.0%) developed MSD	0.0–16.1%	
Total	6	31	37			

Exact McNemar *p* was calculated for the overall paired change in ASEX-defined MSD status among all 37 participants, based on discordant transitions between baseline and follow-up.

**Figure 1:**
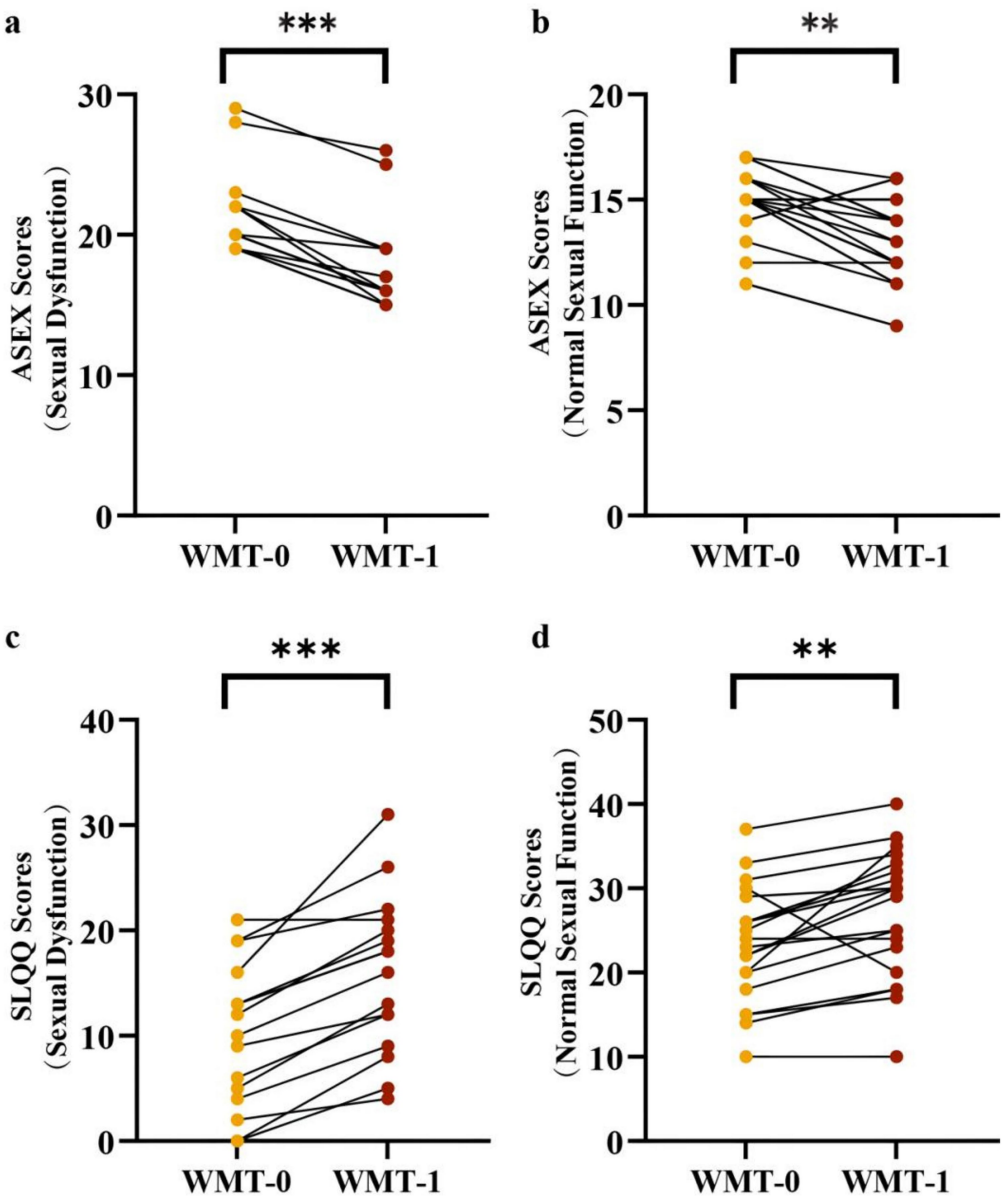
Within-subject changes in ASEX and SLQQ scores from baseline to one-month follow-up. Each dot represents one participant, and lines connect paired measurements before and after WMT. Yellow and red dots denote pre- and post-WMT measurements, respectively. Statistical significance was assessed using paired tests. These paired comparisons were exploratory; *p < 0.05, **p < 0.01, ***p < 0.001. **(a)** Within-subject changes in ASEX scores from baseline to one-month follow-up in patients with baseline MSD. **(b)** Within-subject changes in ASEX scores from baseline to one-month follow-up in participants with normal baseline sexual function. **(c)** Within-subject changes in SLQQ scores from baseline to one-month follow-up in patients with baseline MSD. **(d)** Within-subject changes in SLQQ scores from baseline to one-month follow-up in participants with normal baseline sexual function.

In paired within-subject analyses, ASEX scores were lower at one-month follow-up than at baseline in patients with baseline MSD. A similar within-subject decrease in ASEX scores was observed among participants with normal baseline sexual function. Detailed results are presented in Figures 1a,b and Table 4.

**Table 4:** Within-subject changes in ASEX and SLQQ scores from baseline to one-month follow-up.

Outcome	Group	Before WMT	After 1st WMT	Paired change	95% CI	Standardized paired effect size	*p*	Holm-adjusted *p*
ASEX score	MSD	20.00 (19.00–22.00)	16.00 (15.25–19.00)	−3.75	−4.52 to −2.98	−1.00	<0.001	<0.001
ASEX score	Normal sexual function	15.00 (14.00–16.00)	13.00 (11.00–14.00)	−1.81	−2.62 to −1.00	−0.86	0.0012	0.00241
SLQQ score	MSD	10.13 ± 6.79	15.88 ± 7.47	5.75	3.97 to 7.53	1.72	<0.001	<0.001
SLQQ score	Normal sexual function	22.95 ± 6.89	26.62 ± 7.63	3.67	1.59 to 5.74	0.81	0.0019	0.00241

Data are presented as mean ± SD or median. Paired change was calculated as the follow-up value minus the baseline value. Negative changes in ASEX scores and positive changes in SLQQ scores indicate favorable within-subject changes. Standardized paired effect sizes are presented as numeric values, with the metric selected according to the distribution of paired differences. Raw *p* values were derived from paired within-subject tests, and Holm-adjusted *p* values were calculated across the four ASEX and SLQQ comparisons.

In paired within-subject analyses, SLQQ scores were higher at one-month follow-up than at baseline in patients with baseline MSD and in participants with normal baseline sexual function. These findings indicate favorable within-subject changes in sexual quality-of-life scores over time. Detailed results are presented in Figures 1c,d and Table 4.

### 3.4 Exploratory multivariable sensitivity analyses

In exploratory multivariable sensitivity analyses, the sample-average change was −2.65 points for ASEX scores (95% CI, −3.34 to −1.95) and 4.57 points for SLQQ scores (95% CI, 3.18 to 5.96). After adjustment for the corresponding baseline score, age, baseline depression and anxiety scores, and comorbidity burden, the magnitude of change did not differ significantly between patients with functional gastrointestinal disorders and those with other WMT indications for ASEX scores (*β* = 0.26, 95% CI − 1.07 to 1.59; Holm-adjusted *p* = 1.00) or SLQQ scores (β = −0.91, 95% CI − 4.18 to 2.35; Holm-adjusted *p* = 1.00) (Table 5).

**Table 5:** Multivariable sensitivity analyses of changes in ASEX and SLQQ scores.

Outcome	Adjusted sample-average change	95% CI	β	95% CI	*p*	Holm-adjusted *p*
ΔASEX	−2.65	−3.34 to −1.95	0.26	−1.07 to 1.59	0.691	1.00
ΔSLQQ	4.57	3.18 to 5.96	−0.91	−4.18 to 2.35	0.573	1.00

Models were adjusted for the corresponding baseline score, age, baseline depression and anxiety scores, comorbidity burden, and WMT indication category. HC3 heteroscedasticity-consistent standard errors were used. Positive ΔSLQQ and negative ΔASEX indicate favorable score changes.

### 3.5 Changes in sex hormones in the MSD group before and after the first WMT treatment

None of the normal sexual function group developed sexual dysfunction after the first WMT treatment course. Among the 16 patients with sexual dysfunction, 10 patients (62.50%) no longer met the ASEX-defined sexual dysfunction threshold at one-month follow-up. Although the mean testosterone level decreased from 22.91 ± 9.78 to 21.06 ± 5.80, this change was not statistically significant (*p* = 0.55), and the relatively large standard deviations indicated substantial interindividual variability. Given the small number of patients with baseline MSD (*n* = 16), these analyses were underpowered to detect modest endocrine changes. Therefore, the non-significant hormone findings should be interpreted as inconclusive rather than as evidence that WMT did not affect sex hormone levels. Larger adequately powered studies are needed to clarify whether hormonal mechanisms are involved. Detailed results are presented in Table 6.

**Table 6:** Sex hormone levels before and after WMT in patients with MSD.

Sex Hormone	Before WMT	After 1st WMT	Mean paired change	95% CI	*p*	*q*
Estradiol (pg/mL)	58.65 (44.80–118.77)	59.45 (36.70–85.91)	−10.33	−45.03 to 24.37	0.479	0.829
Prolactin (mIU/L)	292.45 (283.55–388.47)	271.26 (213.88–534.34)	6.60	−193.45 to 206.65	0.936	0.94
Luteinizing hormone (IU/L)	5.19 ± 1.75	4.67 ± 1.55	−0.53	−2.60 to 1.54	0.542	0.829
Follicle-stimulating hormone (IU/L)	7.77 ± 3.97	7.73 ± 3.82	−0.04	−1.10 to 1.03	0.933	0.94
Testosterone (nmol/L)	22.91 ± 9.78	21.06 ± 5.80	−1.85	−9.32 to 5.62	0.553	0.829
Progesterone (ng/mL)	0.47 ± 0.21	0.40 ± 0.12	−0.08	−0.35 to 0.19	0.493	0.829

Data are presented as mean ± SD or median (IQR).

### 3.6 Changes in sex hormones in the normal sexual function group before and after the first WMT treatment

No statistically significant within-subject changes were observed in the six sex hormone parameters among patients with normal baseline sexual function before and after WMT (all *p*-values > 0.05). However, because the study was not powered for endocrine endpoints, the absence of statistically significant hormone changes in this subgroup should be interpreted descriptively and should not be taken as evidence of no hormonal effect. Detailed results are presented in Table 7.

**Table 7:** Sex hormone levels before and after WMT in patients with normal baseline sexual function.

Sex hormone	Before WMT	After 1st WMT	Mean paired change	95%CI	*p*	*q*
Estradiol (pg/mL)	109.31 ± 52.45	86.74 ± 40.87	−22.57	−56.36 to 11.22	0.165	0.495
Prolactin (mIU/L)	344.07 ± 144.72	354.46 ± 169.09	10.39	−60.40 to 81.18	0.721	0.825
Luteinizing Hormone (IU/L)	3.78 (3.03–5.01)	3.23 (2.68–4.72)	−0.49	−1.16 to 0.17	0.128	0.495
Follicle-Stimulating Hormone (IU/L)	4.19 ± 2.10	4.16 ± 2.13	−0.03	−0.34 to 0.28	0.825	0.825
Testosterone (nmol/L)	23.09 ± 6.30	21.74 ± 6.57	−1.35	−6.39 to 3.68	0.558	0.825
Progesterone (ng/mL)	0.34 (0.32–0.51)	0.40 (0.32–0.51)	0.02	−0.09 to 0.12	0.345	0.691

Data are presented as mean ± SD or median (IQR).

### 3.7 Within-subject changes in sexual quality of life from baseline to follow-up according to depressive and anxiety states

**Figure 2:**
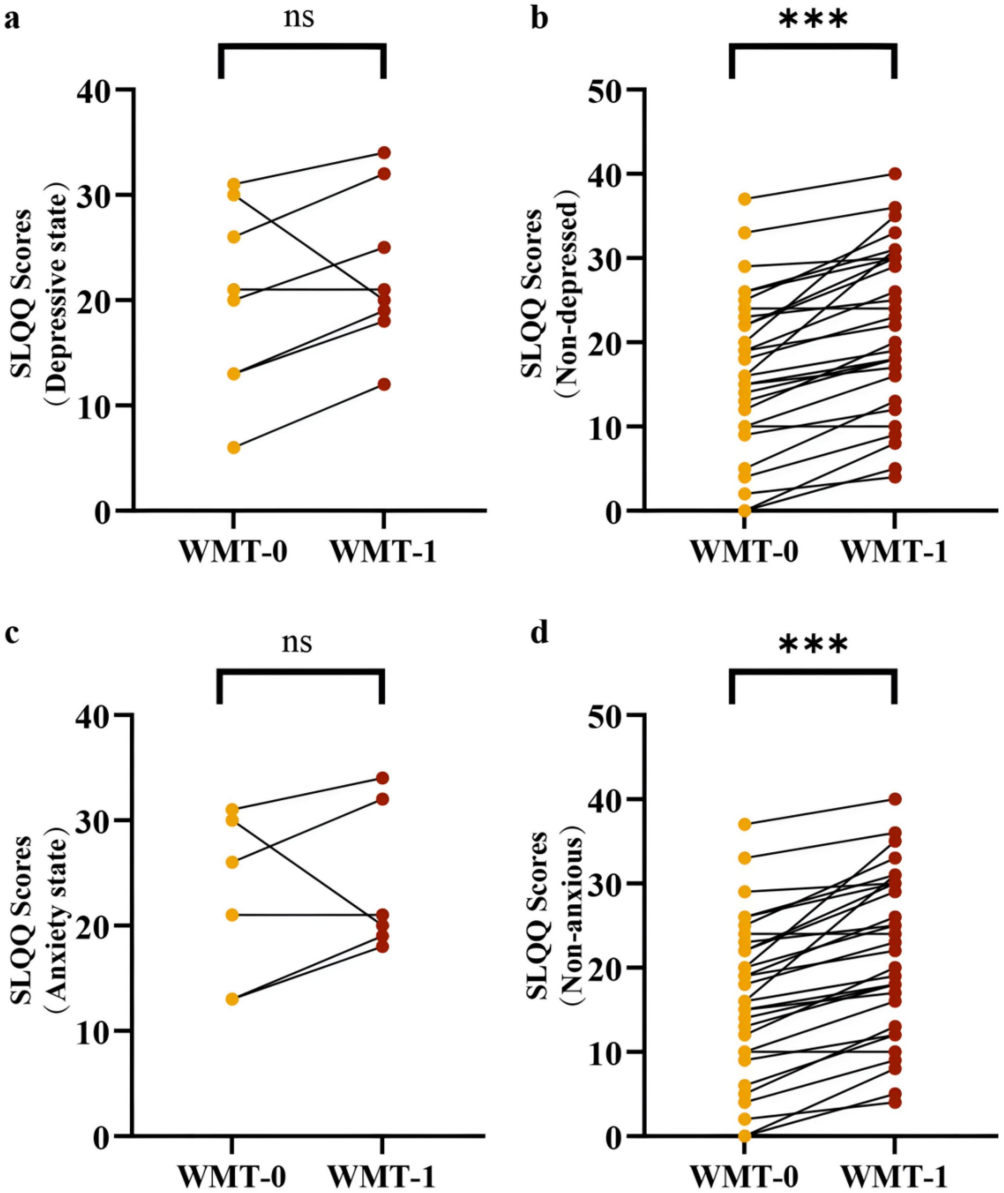
Within-subject changes in SLQQ scores from baseline to one-month follow-up according to depressive and anxiety states. Each dot represents one participant, and lines connect paired measurements before and after WMT. Yellow and red dots denote pre- and post-WMT measurements, respectively. Statistical significance was assessed using paired tests. ns indicates no statistical significance; *p < 0.05, **p < 0.01, ***p < 0.001. **(a)** Comparison of SLQQ scores in patients with depressive state before and after one course of WMT. **(b)** Comparison of SLQQ scores in non-depressed patients before and after one course of WMT. **(c)** Comparison of SLQQ scores in patients with anxiety state before and after one course of WMT. **(d)** Comparison of SLQQ scores in non-anxious patients before and after one course of WMT.

Nine patients with depressive state completed one course of WMT. Their SLQQ scores showed a non-significant upward trend at follow-up compared with baseline (Holm-adjusted *p* = 0.465). Twenty-eight non-depressed patients had higher SLQQ scores at follow-up than at baseline in within-subject analysis (Holm-adjusted *p* < 0.001). Because these subgroup analyses were uncontrolled and exploratory, the observed changes should be interpreted as descriptive within-subject score changes rather than evidence of a WMT effect. Specific results are presented in Figures 2a,b and Table 8.

**Table 8:** Within-subject changes in SLQQ scores after WMT according to depressive and anxiety states.

Psychological state	Before WMT	After 1st WMT	Mean paired change	95% CI	*p*	Holm-adjusted *p*
Depressive state (*n* = 9)	18.33 ± 9.70	21.56 ± 7.60	3.22	−0.97 to 7.41	0.223	0.465
Non-depressed (*n* = 28)	17.11 ± 9.37	22.10 ± 9.79	5.00	3.58 to 6.42	<0.001	<0.001
Anxiety state (*n* = 6)	23.50 (13.00–30.25)	20.50 (18.75–32.50)	1.67	−4.79 to 8.12	0.498	0.498
Non-anxious (*n* = 31)	16.45 ± 9.38	21.58 ± 9.61	5.13	3.83 to 6.42	<0.001	<0.001

Data are presented as mean ± SD or median (IQR).

Six patients with anxiety state completed one course of WMT. Their SLQQ scores did not differ significantly between baseline and follow-up (Holm-adjusted *p* = 0.498). Thirty-one non-anxious patients had higher SLQQ scores at follow-up than at baseline in within-subject analysis (Holm-adjusted *p* < 0.001). These findings should be interpreted as exploratory within-subject observations because the analysis lacked a control group and was not adjusted for potential psychological or clinical confounders. Specific results are presented in Figures 2c,d and Table 8.

### 3.8 Comparative analysis of gut microbial features in sexual dysfunction vs. normal sexually functional patients

**Figure 3:**
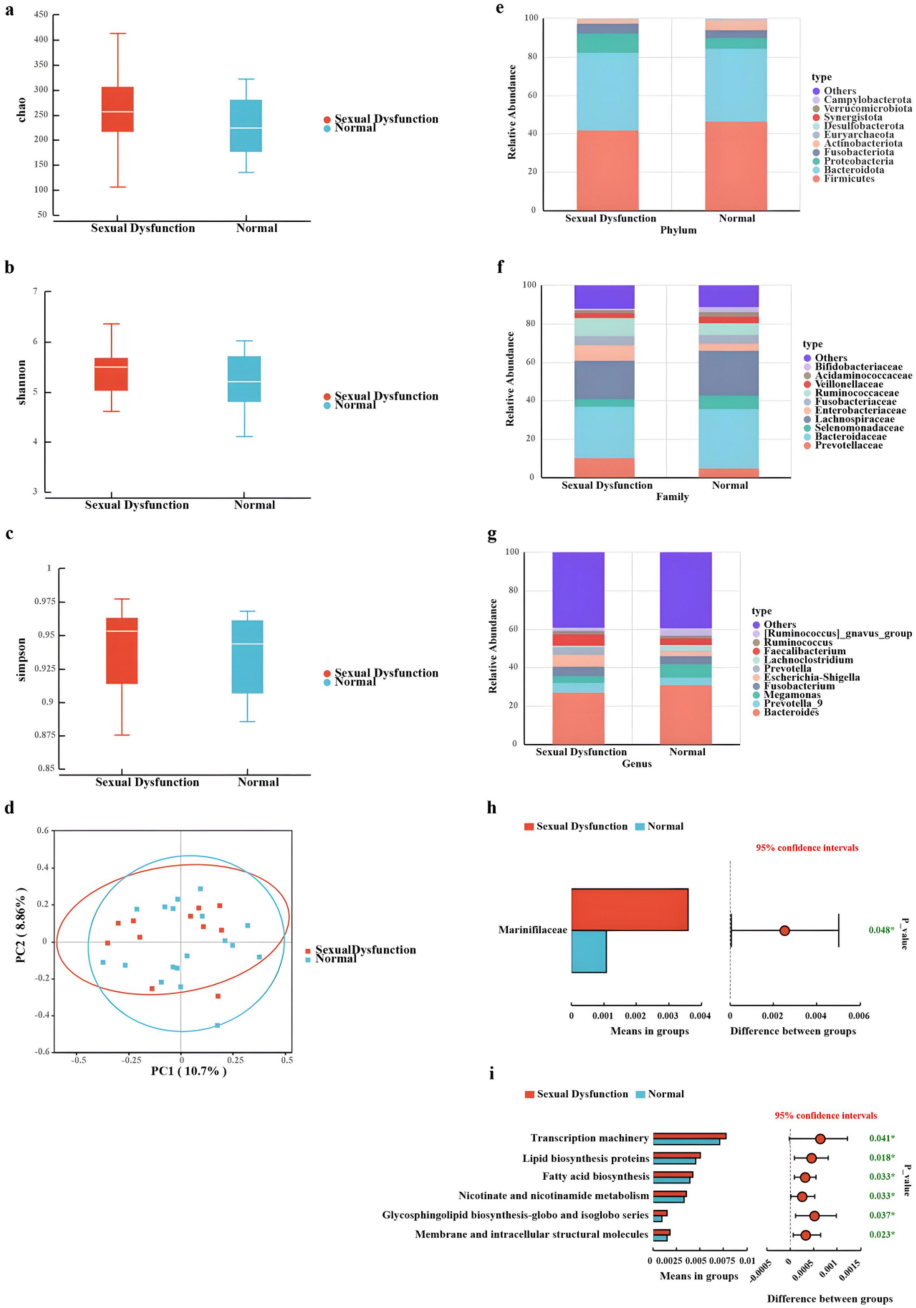
Gut microbiota characteristics between sexual dysfunction and normal sexual function groups. **(a)** Genus-level Chao1 index representing gut microbiota richness in patients with baseline sexual dysfunction and patients with normal baseline sexual function, calculated from 16S rRNA gene sequencing data. **(b)** Genus-level Shannon index reflecting gut microbiota diversity in patients with baseline sexual dysfunction and patients with normal baseline sexual function. **(c)** Genus-level Simpson index reflecting gut microbiota evenness in patients with baseline sexual dysfunction and patients with normal baseline sexual function. **(d)** Principal coordinates analysis of genus-level gut microbiota composition based on Bray–Curtis dissimilarity. **(e)** Comparative analysis of gut microbial composition at the phylum level. Relative abundance is expressed as the percentage of total sequencing reads, and only the most abundant taxa are displayed, with remaining taxa grouped as Others. **(f)** Comparative analysis of gut microbial composition at the family level, presented as relative abundance percentages of total sequencing reads, with less abundant taxa grouped as Others. **(g)** Comparative analysis of gut microbial composition at the genus level, presented as relative abundance percentages of total sequencing reads, with less abundant taxa grouped as Others. **(h)** Comparison of *Marinifilaceae* relative abundance between the MSD group and the normal sexual function group, with effect size shown as the difference between group means and corresponding 95 percent confidence intervals. **(i)** Predicted functional profiles of gut microbiota inferred from 16S rRNA gene data using Tax4Fun, presented as differential abundance of metabolic pathways between groups.

Microbiome-related features were analyzed as secondary exploratory outcomes. Alpha diversity analysis revealed no significant differences in the Chao1, Shannon, or Simpson indices between the sexual dysfunction and normal sexual function groups (*p* > 0.05), indicating that no between-group differences in within-sample richness, diversity, or evenness were detected in this cohort (Figures 3a–c). Principal Coordinate Analysis (PCoA) demonstrated partial overlap in the distribution of samples between groups in the two-dimensional ordination space, with some visual separation across axes (Figure 3d).

At the phylum level, exploratory unadjusted comparisons suggested differences in the relative abundances of *Bacteroidota*, *Firmicutes*, and *Actinobacteriota* between patients with sexual dysfunction and those with normal sexual function (Figure 3e). At the family level, exploratory unadjusted comparisons showed between-group differences in several taxa, including *Selenomonadaceae* and *Bacteroidaceae* (Figures 3f,h). Genus-level analysis suggested differences in the relative abundances of *Bacteroides, Megamonas*, *Lachnoclostridium*, and the *[Ruminococcus] gnavus group* between groups (Figure 3g). Because these taxon-level comparisons were exploratory, involved multiple tests, and were based on a limited number of patients with baseline MSD, these findings are susceptible to both type I errors due to multiple comparisons and type II errors due to insufficient statistical power. Therefore, they should be interpreted cautiously as preliminary microbiome signals rather than confirmed differential taxa.

Tax4Fun-based functional prediction analysis suggested differences in several predicted metabolic pathways between groups, including transcription machinery, lipid biosynthesis proteins, fatty acid biosynthesis, nicotinate and nicotinamide metabolism, glycosphingolipid biosynthesis-globo and isoglobo series, and membrane and intracellular structural molecules (Figure 3i). Because these taxon-level comparisons were exploratory, involved multiple tests, and were based on a limited number of patients with baseline MSD, these findings are susceptible to both type I errors due to multiple comparisons and type II errors due to insufficient statistical power. Several taxa identified in this cohort, including *Bacteroides*, *Lachnoclostridium*, *Megamonas*, and *the [Ruminococcus] gnavus group*, showed partial overlap with ED-related microbiota signals reported in previous studies. Specifically, *Bacteroides*, *Lachnoclostridium*, and *Megamonas* were reported to be enriched in ED patients, whereas *Ruminococcus gnavus* was also reported to be increased in another ED pilot study (Qiao et al., 2024; Su et al., 2025). Therefore, these findings should be interpreted cautiously as preliminary relative-abundance signals observed in the analyzed cohort rather than confirmed ED-specific microbial signatures.

### 3.9 Within-subject gut microbiota changes before and after WMT in all patients

**Figure 4:**
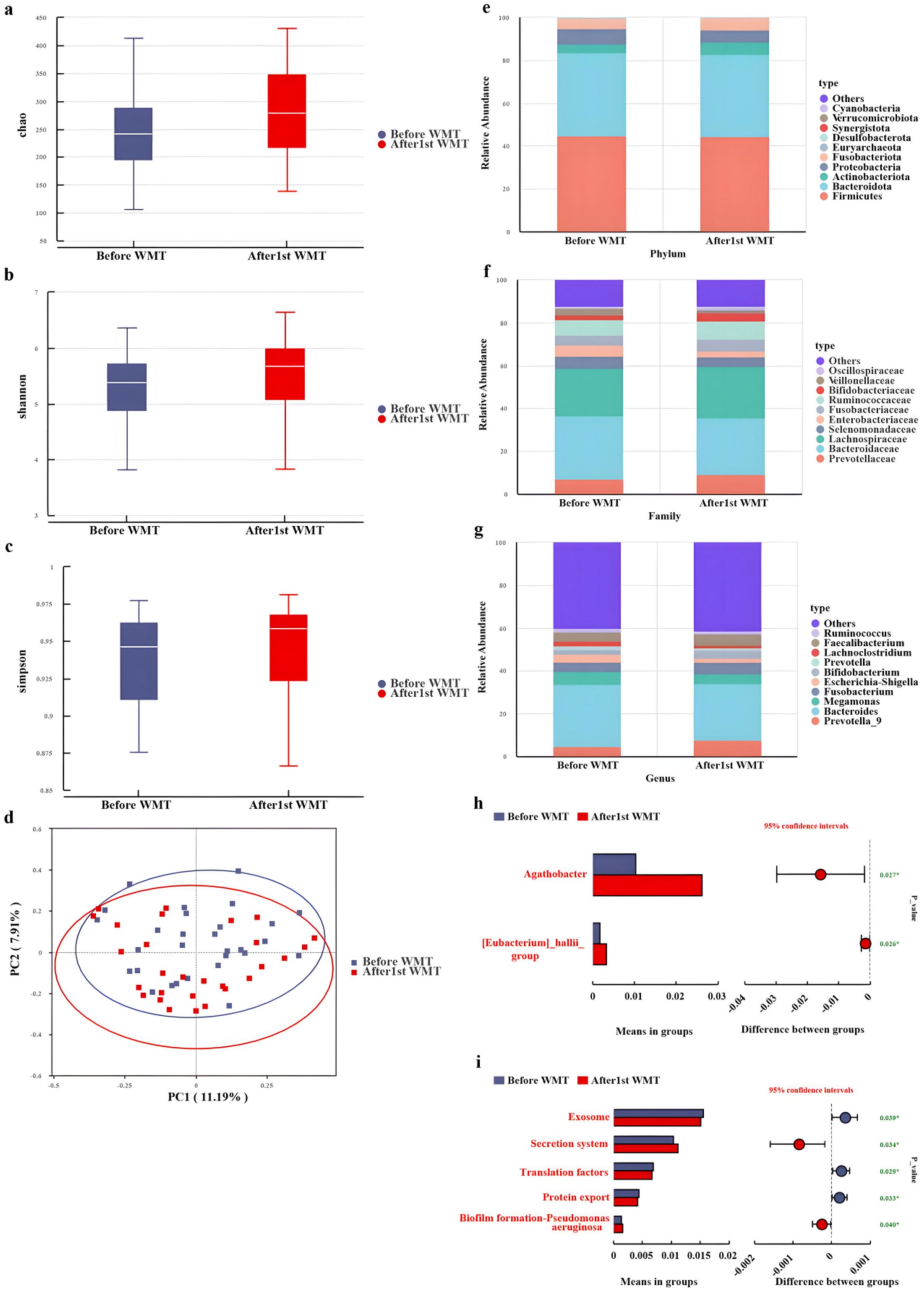
Gut microbiota characteristics in all patients before and after the first WMT. **(a)** Genus-level Chao1 index reflecting gut microbiota richness in all patients before WMT and after the first WMT session, calculated from 16S rRNA gene sequencing data. **(b)** Genus-level Shannon index reflecting gut microbiota diversity in all patients before WMT and after the first WMT session. **(c)** Genus-level Simpson index reflecting gut microbiota evenness in all patients before WMT and after the first WMT session. **(d)** Principal coordinates analysis of genus-level gut microbiota composition before WMT and after the first WMT session, based on Bray–Curtis dissimilarity, with overall compositional differences assessed using permutational multivariate analysis of variance. **(e)** Comparative analysis of gut microbial composition at the phylum level before WMT and after the first WMT session. Relative abundance is expressed as the percentage of total sequencing reads. Only the most abundant taxa are displayed, and remaining taxa are grouped as Others. **(f)** Comparative analysis of gut microbial composition at the family level before WMT and after the first WMT session, presented as relative abundance percentages of total sequencing reads, with remaining taxa grouped as Others. **(g)** Comparative analysis of gut microbial composition at the genus level before WMT and after the first WMT session, presented as relative abundance percentages of total sequencing reads, with remaining taxa grouped as Others. **(h)** Comparison of *Agathobacter* and *the Eubacterium hallii* group relative abundance before WMT and after the first WMT session, with effect size shown as the difference between paired group means and corresponding 95 percent confidence intervals. **(i)** Predicted functional profiles inferred from 16S rRNA gene data using Tax4Fun, presented as differential abundance of metabolic pathways before WMT and after the first WMT session.

Within-subject comparisons of gut microbiota characteristics before and after WMT treatment across all patients revealed an upward trend in alpha diversity metrics post-WMT, including the Chao1 index (species richness), Shannon index (community diversity), and Simpson index (community evenness). However, these changes did not reach statistical significance (*p* > 0.05) (Figures 4a–c). PCoA demonstrated partial overlap but incomplete clustering between pre- and post-WMT microbial communities at the genus level, indicating discernible structural variations in gut microbiota composition following the first WMT cycle (Figure 4d).

Changes in relative abundance were observed across multiple taxonomic levels between baseline and one-month follow-up. At the phylum level, *Euryarchaeota* and *Proteobacteria* exhibited increased relative abundances, while *Firmicutes* and other minor phyla demonstrated modest reductions (Figure 4e). Family-level analysis revealed decreased relative abundances of *Veillonellaceae* and *Enterobacteriaceae*, with varying degrees of enrichment observed in other bacterial families (Figure 4f). Genus-level profiling indicated declines in *Ruminococcus*, *Lachnoclostridium*, *Prevotella*, *Escherichia-Shigella*, *Megamonas*, and *Bacteroides*, alongside heterogeneous increases in other genera (Figure 4g). Following WMT intervention, exploratory unadjusted taxon-level analysis showed increases in the relative abundances of *Agathobacter* and the *[Eubacterium] hallii group*, two taxa associated with butyrate production and gut health (Figure 4h). Because these comparisons were not corrected for multiple testing, these changes should be interpreted cautiously as exploratory microbiome signals rather than definitive WMT-induced taxonomic alterations. Tax4Fun-based analysis of predicted functional profiles showed differences in pathway-level signals between pre- and post-WMT samples. After WMT, pathways including Amino Acid Metabolism and Lipid Metabolism showed higher predicted relative abundance, whereas pre-WMT samples showed higher predicted signals for pathways such as Cell Motility and Signal Transduction (Figure 4i). To improve visualization of WMT-associated microbiota composition, genus-level heatmaps were additionally generated for all patients and for the sexual dysfunction subgroup. These heatmaps illustrated the relative abundance patterns of dominant genera before and after the first WMT and complemented the stacked bar plots and beta-diversity PCoA analyses (Supplementary Figures S1a,b).

### 3.10 Within-subject gut microbiota changes before and after WMT in patients with baseline sexual dysfunction

**Figure 5:**
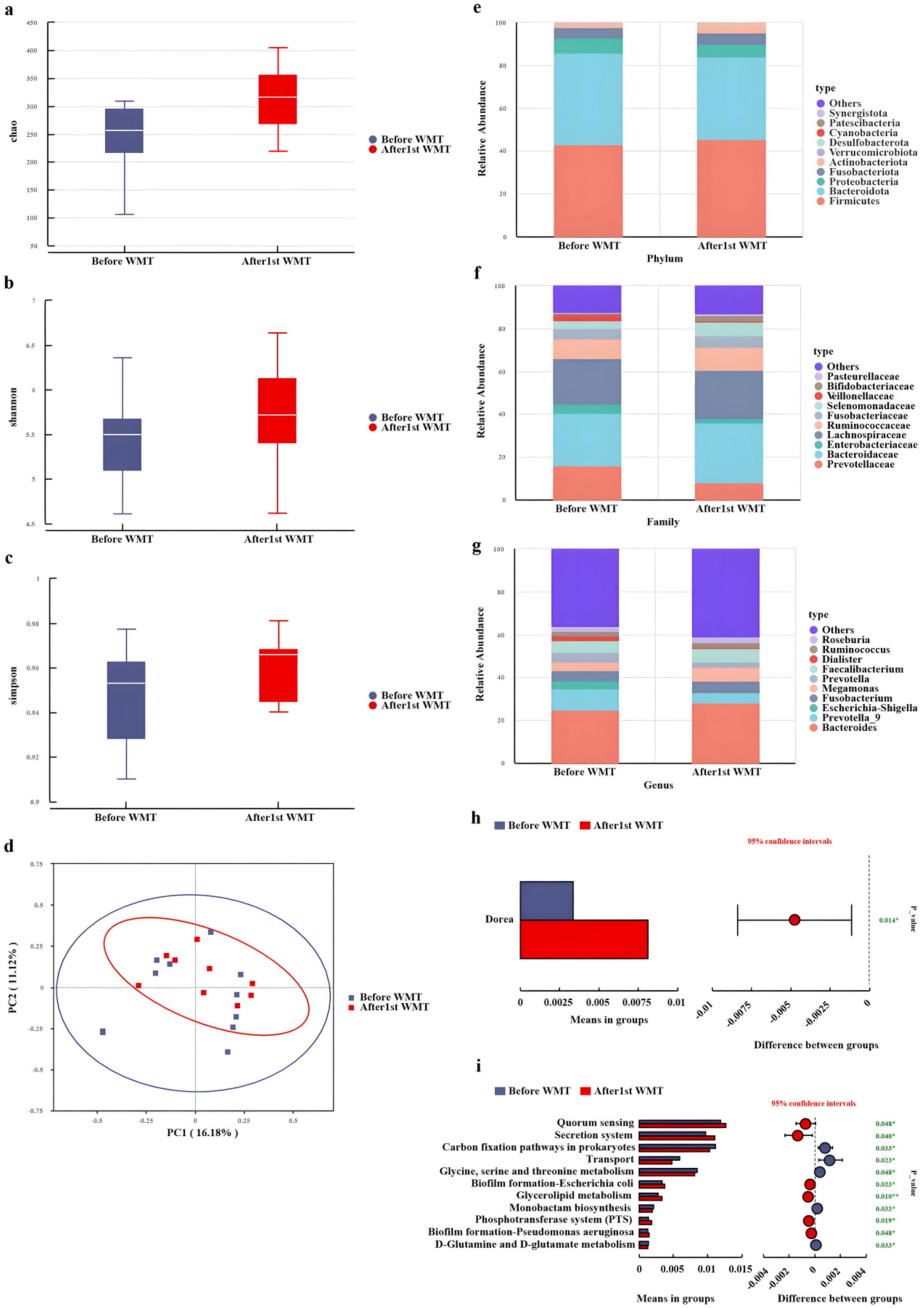
Gut microbiota characteristics in patients with sexual dysfunction before and after the first WMT. **(a)** Genus-level Chao1 index reflecting gut microbiota richness in patients with sexual dysfunction before WMT and after the first WMT session, calculated from *16S rRNA* gene sequencing data. **(b)** Genus-level Shannon index reflecting gut microbiota diversity in patients with sexual dysfunction before WMT and after the first WMT session. **(c)** Genus-level Simpson index reflecting gut microbiota evenness in patients with sexual dysfunction before WMT and after the first WMT session. **(d)** Principal coordinates analysis of genus-level gut microbiota composition before WMT and after the first WMT session, based on Bray–Curtis dissimilarity, with overall compositional differences assessed using permutational multivariate analysis of variance. **(e)** Comparative analysis of gut microbial composition at the phylum level before WMT and after the first WMT session. Relative abundance is expressed as the percentage of total sequencing reads. Only the most abundant taxa are displayed, and remaining taxa are grouped as Others. **(f)** Comparative analysis of gut microbial composition at the family level before WMT and after the first WMT session, presented as relative abundance percentages of total sequencing reads, with remaining taxa grouped as Others. **(g)** Comparative analysis of gut microbial composition at the genus level before WMT and after the first WMT session, presented as relative abundance percentages of total sequencing reads, with remaining taxa grouped as Others. **(h)** Comparison of *Dorea* relative abundance before WMT and after the first WMT session, with effect size shown as the difference between time points and corresponding 95 percent confidence intervals. **(i)** Predicted functional profiles inferred from *16S rRNA* gene data using Tax4Fun, presented as differential abundance of metabolic pathways before WMT and after the first WMT session.

Within-subject comparisons of gut microbiota characteristics in patients with baseline sexual dysfunction showed upward trends in alpha diversity metrics at follow-up, including the Chao1 index, Shannon index, and Simpson index. However, these changes did not reach statistical significance (*p* > 0.05) (Figures 5a–c). PCoA showed partial clustering of baseline and follow-up gut microbiota samples, with incomplete overlap in microbial community distribution (Figure 5d). These findings suggest possible compositional differences over time, despite the absence of significant alpha-diversity changes.

Taxonomic shifts in gut microbiota composition were observed across hierarchical levels between baseline and follow-up. At the phylum level, the relative abundances of *Firmicutes*, *Actinobacteriota*, and *Fusobacteriota* were higher at follow-up (Figure 5e). Family-level analysis revealed elevated relative abundances of *Bacteroidaceae*, *Ruminococcaceae*, *Lachnospiraceae*, *Selenomonadaceae*, and *Fusobacteriaceae* (Figure 5f). Genus-level profiling demonstrated increased abundances of *Bacteroides*, *Megamonas*, and *Roseburia* (Figure 5g). In patients with sexual dysfunction, exploratory unadjusted analysis showed an increase in the relative abundance of *Dorea* after WMT (Figure 5h). This change should be regarded as a hypothesis-generating microbiome signal rather than evidence of a causal or mechanistic role, particularly because the subgroup analysis was based on only 16 patients and the taxon-level comparisons were not adjusted for multiple testing.

Comparative analysis of predicted microbial metabolic pathways in patients with baseline sexual dysfunction showed higher predicted signals for Quorum Sensing, Secretion System, Glycerolipid Metabolism, and Phosphotransferase System pathways at follow-up. Baseline samples showed higher predicted signals for pathways such as Carbon Fixation Pathways in Prokaryotes, Transport, and Glycine, Serine and Threonine Metabolism (Figure 5i). These functional predictions should be interpreted cautiously because they were inferred from 16S rRNA gene sequencing data and were not validated by shotgun metagenomics or metabolomics, and cannot establish true functional changes or mechanisms.

### 3.11 Correlation between SLQQ/ASEX scores, sex hormones, and gut microbiota abundance

**Figure 6:**
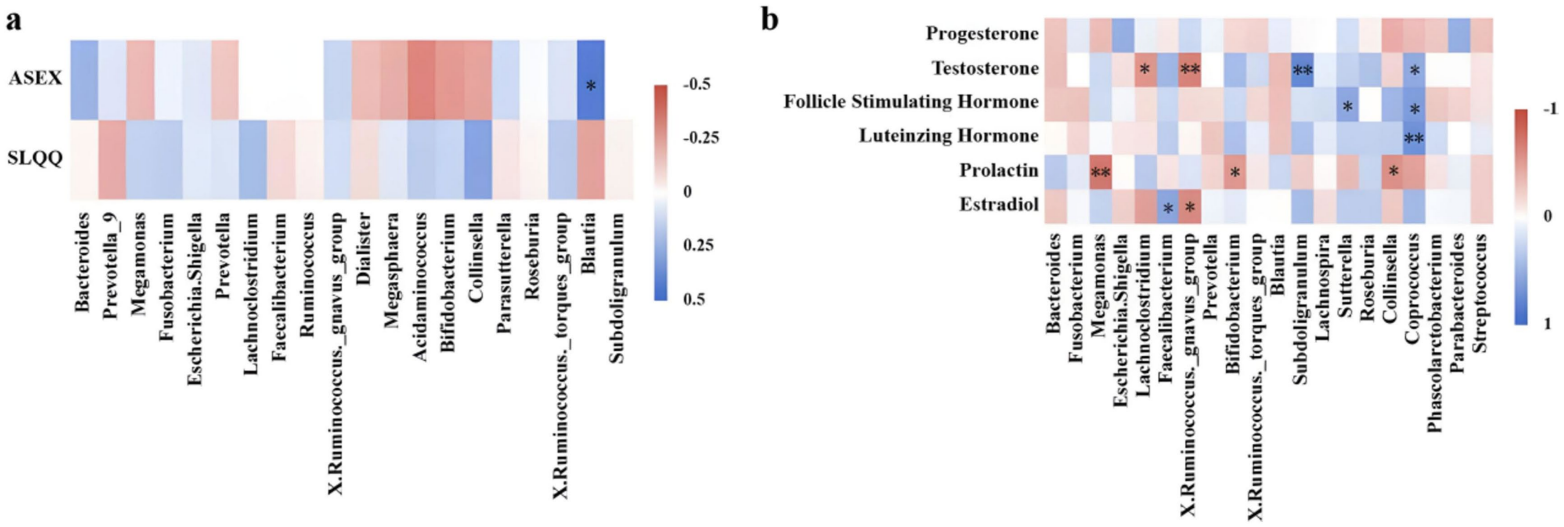
Correlation between SLQQ/ASEX scores, sex hormone, and genus-level gut microbiota abundance. Correlations were assessed using Spearman’s rank correlation. *p < 0.05, **p < 0.01. Colors represent Spearman correlation coefficients, with blue indicating positive correlations and red indicating negative correlations. **(a)** Heatmap showing correlations between ASEX and SLQQ scores and genus-level gut microbiota relative abundance. **(b)** Heatmap showing correlations between circulating sex hormones and genus-level gut microbiota relative abundance, based on Spearman rank correlation analysis. For both panels, gut microbiota abundance refers to genus-level relative abundance derived from 16S rRNA gene sequencing data. *p < 0.05, **p < 0.01. These correlations are exploratory and unadjusted and should be interpreted as hypothesis-generating rather than evidence of causal relationships.

Exploratory Spearman correlation analyses were performed to evaluate potential associations between sexual-function scores, sex hormone levels, and genus-level gut microbiota abundance. At the genus level, the abundance of *Subdoligranulum* exhibited a significant positive correlation with ASEX scores (*p* < 0.05) (Figure 6a). At the genus level, testosterone exhibited negative correlations with *Lachnoclostridium* and the *[Ruminococcus] gnavus group*, while demonstrating positive correlations with *Subdoligranulum* and *Coprococcus*. Follicle-stimulating hormone (FSH) showed positive correlations with *Sutterella* and *Coprococcus*, and luteinizing hormone (LH) was similarly positively correlated with *Coprococcus*. Prolactin displayed negative correlations with *Megamonas*, *Bifidobacterium*, and *Collinsella*. Estradiol, conversely, exhibited a positive correlation with *Faecalibacterium* but a negative correlation with the *[Ruminococcus] gnavus group*. However, these analyses were not adjusted for multiple comparisons and were not controlled for potential confounders such as age, comorbidities, psychological status, or baseline microbiota structure. Therefore, these associations should be interpreted as exploratory and hypothesis-generating rather than evidence of independent, predictive, or causal relationships (Figure 6b).

These heatmap-based analyses illustrate exploratory, unadjusted associations between sexual-function scores, circulating sex hormones, and gut microbiota at the genus level. Given the absence of multivariable adjustment for age, comorbidities, or baseline microbiota structure, these findings should be interpreted as hypothesis-generating rather than evidence of independent or causal relationships.

## 4 Discussion

In this exploratory real-world single-arm pre–post observational cohort, sexual-function and sexual quality-of-life scores showed favorable within-subject changes at one-month follow-up among men receiving WMT. In this exploratory real-world single-arm pre–post observational cohort, sexual-function and sexual quality-of-life scores showed favorable within-subject changes at one-month follow-up among men receiving WMT. Specifically, 62.5% of patients with baseline sexual dysfunction no longer met the ASEX-defined sexual dysfunction threshold at 1-month follow-up, accompanied by lower ASEX scores and higher SLQQ scores. However, because the study was non-randomized and lacked a placebo, sham, untreated, or standard-care control group, these findings should be interpreted as preliminary associations rather than evidence of a causal therapeutic effect of WMT. In particular, sexual-function outcomes are highly susceptible to placebo and expectancy effects, regression to the mean, natural symptom fluctuation, and unmeasured psychosocial or clinical confounders.

These findings should be considered within the broader context of MSD as a heterogeneous and multifactorial condition. Because ED is one of the most prevalent and best-characterized MSD subtypes, ED-focused studies provide useful mechanistic context. Previous studies have shown that ED may improve after correction of systemic disease states, such as kidney transplantation in patients with end-stage renal disease (Kang et al., 2020). Diabetes-related ED studies have further highlighted the roles of endothelial dysfunction, microvascular injury, oxidative stress, inflammation, fibrosis, and tissue regeneration, with stem cell therapy, microRNA regulation, and inducible nitric oxide synthase inhibition being explored as emerging therapeutic directions (Kang et al., 2022; Wang et al., 2022; Liu et al., 2021). Compared with these molecularly targeted or regenerative strategies, WMT represents a microbiota-based intervention that may be related to gut microbial ecology. Whether WMT influences metabolic, inflammatory, endocrine, or neurovascular pathways in MSD remains unknown and requires direct functional validation.

Previous ED-focused microbiome studies provide an important context for interpreting our findings. Recent studies have reported altered gut microbiota composition in patients with ED and suggested that gut dysbiosis may contribute to ED through effects on intestinal barrier function, systemic inflammation, cardiovascular regulation, metabolic disturbance, mental health, and gut–brain axis signaling (Chen et al., 2026). Recent studies using observational or Mendelian randomization approaches have also suggested associations between specific gut microbial taxa and ED risk (Zhang et al., 2024; Okamoto et al., 2020). Our study is consistent with these reports in showing differences in gut microbial composition between patients with sexual dysfunction and those with normal sexual function. However, unlike most previous ED microbiome studies, the present study further explored microbiota changes before and after WMT in a real-world clinical cohort. Therefore, our findings add preliminary clinical evidence to the gut microbiota–sexual health hypothesis, but they should not be interpreted as confirming specific microbial biomarkers or causal mechanisms.

The present study should also be interpreted in the context of microbiota-based therapies. WMT is a standardized form of fecal microbiota transplantation designed to improve the quality control and safety of microbiota preparation (Shi, 2020). The Nanjing consensus provides methodological guidance for WMT, and microbiota transplantation has been explored in gastrointestinal, inflammatory, and metabolic disorders (Zhang et al., 2026). However, direct clinical evidence for WMT in MSD remains limited. Therefore, our study differs from previous microbiota-based therapy studies by focusing on sexual-function outcomes, while sharing the broader therapeutic rationale of restoring microbial ecological balance. Randomized controlled trials are still required to determine whether WMT has a true therapeutic effect on MSD.

From a mechanistic perspective, gut microbiota may plausibly influence sexual function through several interconnected pathways. Microbial metabolites such as short-chain fatty acids may regulate intestinal barrier integrity, immune responses, systemic inflammation, metabolic homeostasis, and vascular function (Chen et al., 2026). In our cohort, sex hormone levels did not change significantly after WMT. However, because only 16 patients with baseline MSD were included and no formal power calculation was performed for endocrine endpoints, these null findings are inconclusive and should not be interpreted as evidence against hormonal involvement. Hormonal mechanisms therefore cannot be excluded and require evaluation in larger adequately powered studies. Non-hormonal pathways, including inflammation (Liu et al., 2022), endothelial nitric oxide signaling (Zhu et al., 2023) and metabolic regulation (Ahmad et al., 2023) may also contribute to sexual-function changes, but these mechanisms remain speculative in the absence of direct metabolomic, inflammatory-marker, or functional validation.

In relation to specific microbial features, our exploratory analyses suggested several taxonomic changes after WMT. Across all participants, WMT was accompanied by changes in several taxa, including *Agathobacter* and *Anaerobutyricum*, while the MSD group showed a change in *Dorea*. Some of these taxa have been associated with short-chain fatty acid production, host metabolic regulation, intestinal barrier function, or gut–brain signaling in previous studies. However, our 16S rRNA sequencing data cannot determine strain-level identity, microbial functional activity, or metabolite production. Given the small sample size, exploratory nature of the microbiota analyses, lack of comprehensive multiple-comparison correction, and absence of metagenomic or metabolomic validation, these taxonomic findings should be interpreted only as hypothesis-generating microbial signals rather than confirmed biomarkers or mechanistic mediators of sexual-function changes.

We also observed a higher relative abundance of *Marinifilaceae* in the MSD group. Some genera assigned to or related to *Marinifilaceae*, such as *Butyricimonas*, have been reported to include butyric acid-producing species, and SCFA-producing bacteria have been implicated in intestinal barrier integrity, immune regulation, inflammation, and metabolic homeostasis in previous studies (Chi et al., 2019). However, we did not perform species-level identification, shotgun metagenomics, targeted SCFA measurement, metabolomics, or functional assays. Therefore, the observed increase in *Marinifilaceae* should be interpreted as an exploratory microbial association with MSD rather than evidence of altered SCFA production, inflammation, endothelial signaling, or causal microbial mechanisms. In this study, we further explored sexual-function score changes and gut microbiota profiles before and after WMT in men with baseline sexual dysfunction, providing preliminary data for future controlled studies on environmental factors (Wu et al., 2021c), gut microbiota (Wu et al., 2021a; Wu et al., 2022c; Wu et al., 2025), metabolic biomarkers (Wu et al., 2021b; Wu et al., 2023), and the influence of WMT (Lin et al., 2024; He et al., 2024; Liang et al., 2024; Hu et al., 2024; Lin et al., 2025; Wu et al., 2022b; Wu et al., 2022a) in male sexual health.

Regarding specific microbial compositions, previous ED-related microbiome studies have reported altered abundances of several taxa, including enrichment of Actinomyces and depletion of *Coprococcus_1*, *Lachnospiraceae_FCS020_group*, *Lactococcus*, *Ruminiclostridium_5*, and *Ruminococcaceae_UCG_002* in ED patients (Kang et al., 2024). Another pilot study reported decreased *Bacteroides intestinalis* and increased *Ruminococcus gnavus* in ED samples, while Mendelian-randomization evidence suggested possible associations of *Lachnospiraceae*-related taxa and several genera with ED risk (Zhang et al., 2024). In the present cohort, the main genus-level relative-abundance differences between patients with baseline MSD and those with normal baseline sexual function involved *Bacteroides*, *Megamonas*, *Lachnoclostridium*, and the *[Ruminococcus] gnavus group* (Qiao et al., 2024). Thus, our findings showed only partial overlap with previously reported ED-associated taxa, mainly involving the *[Ruminococcus] gnavus group* and *Lachnospiraceae*-related signals. In contrast, taxa such as *Actinomyces*, *Coprococcus_1*, *Ruminiclostridium_5*, and *Ruminococcaceae_UCG_002* were not prominent differential taxa in our analysis, and genus-level *Bacteroides* in our 16S data cannot be equated with species-level *Bacteroides intestinalis* (Su et al., 2025). Therefore, the apparent prevalence of these taxa in the analyzed patients should be interpreted as exploratory relative-abundance patterns rather than evidence of ED-specific microbial composition.

This study has several limitations. First, this was a non-randomized, single-arm observational pre–post study without a placebo or standard-care control arm. Given the well-recognized placebo response and expectancy effects in sexual dysfunction, the observed improvements in ASEX and SLQQ may partly reflect placebo effects, regression to the mean, natural symptom fluctuation, or concurrent care rather than a definitive causal effect of WMT. Sexual-function outcomes are also susceptible to psychological confounders, including mood changes, relationship factors, and psychosocial stressors, which were not fully controlled in the present study design. In addition, the normal baseline sexual-function group should not be considered a true control group because these participants also received WMT and differed clinically from the MSD group at baseline. Therefore, comparisons involving this group cannot estimate the treatment effect of WMT. Second, an additional major limitation was the substantial clinical heterogeneity of the cohort. Participants received WMT for diverse indications, including functional gastrointestinal, inflammatory or immune-mediated, metabolic, neuropsychiatric, neurological, and oncology-related conditions, rather than specifically for MSD. These treated diseases may independently influence sexual function through symptom burden, pain, fatigue, systemic inflammation, metabolic or vascular dysfunction, endocrine abnormalities, psychological distress, medication exposure, and impaired quality of life. At the same time, these conditions may also shape gut microbiota composition. Therefore, the observed microbiome–sexual-function associations may reflect disease-specific dysbiosis, indication-related bias, concurrent care, or changes in underlying disease activity rather than microbiota features specifically attributable to MSD. Although we performed exploratory multivariable sensitivity analyses adjusting for available baseline variables, detailed information on disease severity, disease subtype, indication-specific disease activity, concurrent supportive care, and changes in co-interventions was not sufficiently captured. The small sample size also required broad grouping of indications and precluded comprehensive indication-specific adjustment. Consequently, substantial residual and unmeasured confounding remains possible, and the observed within-subject changes in sexual-function scores and microbiota profiles cannot be attributed specifically or causally to WMT. Third, the number of patients with sexual dysfunction was modest (*n* = 16), which limits statistical power and may restrict the generalizability of the findings. Fourth, the study may be underpowered to detect modest changes in sex hormones; therefore, non-significant hormone findings should not be interpreted as evidence of no hormonal involvement. Fifth, multiple taxon-level comparisons and microbiome–clinical correlation analyses were conducted in this study. Although these analyses were treated as exploratory, formal multiple-comparison correction was not applied to all microbiome-related tests; therefore, some associations based on unadjusted *p*-values may represent false-positive findings. The observed associations between specific taxa and sexual-function measures should therefore be regarded as exploratory and hypothesis-generating. In addition, because our 16S rRNA gene sequencing data mainly supported genus-level relative-abundance comparisons, patient-level prevalence and species-level identification of ED-associated taxa could not be reliably established. Future studies with larger sample sizes, independent validation cohorts, and appropriate false discovery rate correction are needed to confirm these microbial signals. Sixth, the study had limited mechanistic depth because the microbiome analysis was based on 16S rRNA gene sequencing and Tax4Fun-based functional inference. These approaches characterize taxonomic composition and predicted pathway profiles, but they do not directly measure microbial genes, transcripts, metabolites, or host functional responses. We did not perform shotgun metagenomic sequencing, metatranscriptomic profiling, metabolomic analysis, targeted short-chain fatty acid quantification, inflammatory-marker assessment, endothelial nitric oxide or eNOS pathway evaluation, or experimental validation in animal or cellular models. Therefore, the observed taxonomic differences, predicted functional pathways, and microbiota–clinical correlations cannot establish true microbial functional changes or microbiota-mediated mechanisms. No conclusions can be drawn regarding SCFA production, inflammatory signaling, endothelial nitric oxide signaling, or other functional pathways linking gut microbiota to sexual-function changes. Future studies should integrate multi-omics profiling, microbial metabolite measurements, host immune and endothelial markers, and mechanistic experimental models to determine whether the exploratory microbiome signals observed in this study are biologically meaningful. Finally, the follow-up duration was limited to one month after the first WMT session, which is insufficient to determine whether the observed changes in sexual-function scores or gut microbiota profiles are durable, transient, or clinically meaningful over the long term. Therefore, the short follow-up period represents an important limitation of this study. Collectively, these limitations warrant cautious interpretation of causal inference. Future studies should incorporate randomized controlled designs with at least gut-healthy control group, a larger sample size, longer follow-up, broader assessment of ED, psychological well-being, relationship status and the influence of concomitant metabolic diseases.

## 5 Conclusion

In this exploratory single-arm pre–post observational study, favorable within-subject changes in sexual-function and sexual quality-of-life scores were observed after WMT in men with baseline MSD. These findings suggest a possible association between WMT, gut microbiota modulation, and male sexual health. However, because the study lacked an appropriate control arm, these findings cannot establish therapeutic efficacy and should be interpreted only as preliminary associations. Serum hormone and microbiome findings were secondary exploratory outcomes and should be regarded as hypothesis-generating. Randomized placebo- or sham-controlled studies with larger sample sizes, longer follow-up and repeated assessments, and better control of psychosocial and clinical confounders are needed to determine whether WMT is causally associated with sustained improvements in MSD.

## Data Availability

The datasets presented in this study can be found in online repositories. The names of the repository/repositories and accession number(s) can be found at: https://www.ncbi.nlm.nih.gov/, PRJNA1307230.
