# Supplementary material for: Changes in sexual-function scores after washed microbiota transplantation in men with sexual dysfunction: an exploratory single-arm pre–post observational study
Source: Front Microbiol. 2026 Aug 5;17:1906680. doi: 10.3389/fmicb.2026.1906680 (PMC13485940; doi:10.3389/fmicb.2026.1906680)
Supplement: Supplementary file 1 [file Image_1.pdf]

Supplementary Figure

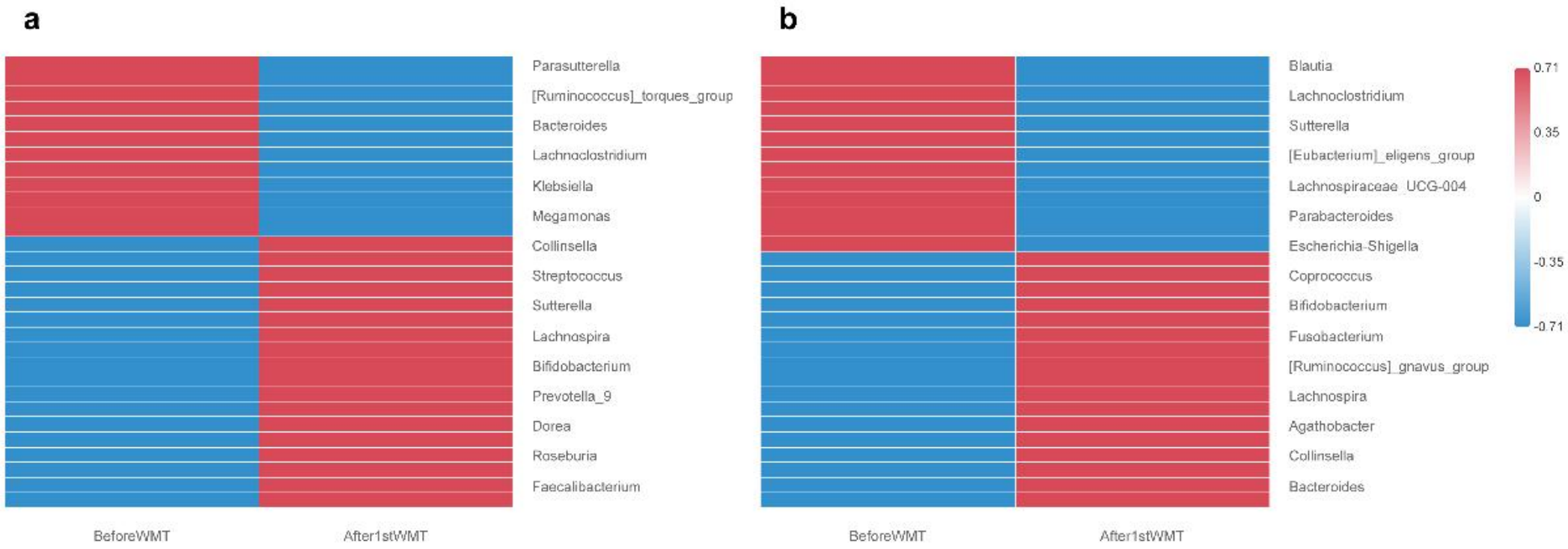

Supplementary Figure S1. Genus-level heatmaps showing gut microbiota composition before and after the first WMT.

(a) Heatmap of the dominant genera in all patients before WMT and after the first WMT.

(b) Heatmap of the dominant genera in patients with sexual dysfunction before WMT and after the first WMT.

Rows represent bacterial genera, and columns represent the Before WMT and After1stWMT groups. Colors indicate scaled relative abundance, with red indicating relatively higher abundance and blue indicating relatively lower abundance. The heatmaps were used to visualize overall genus-level compositional patterns following WMT.
